# Primary Spontaneous Bilateral Pneumothorax in a Neonate

**Published:** 2014-09-01

**Authors:** Kamaldeep Arora, Shasanka Shekhar Panda, Rashmi Ranjan Das, Pankaj Kumar Mohanty, Meely Panda

**Affiliations:** 1 Department of Pediatrics, Dayanand Medical College and Hospital, Ludhiana, Punjab, 141001, India; 2 Department of Pediatric Surgery, All India Institute of Medical Sciences (AIIMS),New Delhi-110029, India; 3 Department of Pediatrics, All India Institute of Medical Sciences (AIIMS), Bhubaneswar-751019, India; 4 Department of Neonatology, Manipal Hospital, Bangalore- 560017, India; 5 Department of Community Medicine, Pandit Bhagwat Dayal Sharma Post Graduate Institute of Medical Sciences, Rohtak, Haryana, 124001, India

**Keywords:** Intercostal drain, Newborn, Oxygen supplementation, Spontaneous pneumothorax

## Abstract

Pneumothorax, though rare, is a recognized cause of respiratory distress in the immediate newborn period. It may occur spontaneously or secondary to various underlying lung diseases. Here we share our experience of a neonate with spontaneous pneumothorax with mild to moderate respiratory distress, who recovered completely with conservative management with an oxygen-enriched atmosphere and no surgical intervention.

## CASE REPORT

A 3350 g female neonate was born at term gestation by caesarean section to a primigravida mother after an uneventful antenatal period. There was no history of trauma during delivery or meconium stained liquor. She cried immediately after birth and did not require any resuscitation. At 30 min of life, she developed tachypnea with grunting and cyanosis (SpO2 varying from 80 to 83%). Supplemental oxygen via hood (FiO2 40%) was started after which cyanosis resolved (SpO2 increased to 88-90%), but respiratory distress persisted. Physical examination revealed a heart rate of 156/min, capillary refill time of 3 seconds, good volume pulses, and respiratory rate of 68/min. Breath sounds were decreased bilaterally without any crepitations or wheeze. Rest of the systemic examination was within normal limits. A chest x-ray was ordered. Arterial blood gas measurement (while the neonate was on 40% FiO2) showed combined respiratory with metabolic acidosis without hypoxemia (pH = 7.2, PaCO2 = 62 mmHg, PaO2 = 70 mmHg, HCO3 = 19.6, and base excess = -8.0). Meanwhile, the chest radiograph revealed bilateral pneumothoraces with normal pulmonary vasculature and normal cardiac silhouette (Fig. 1). Sepsis screen (total leucocyte count = 11500/mm3, absolute neutrophils count = 5600, band cell count = 0, I:T ratio = 0.1, micro-ESR = 5mm in 1st hour, CRP = 0.5 mg/dL), and blood culture were negative.

**Figure:**
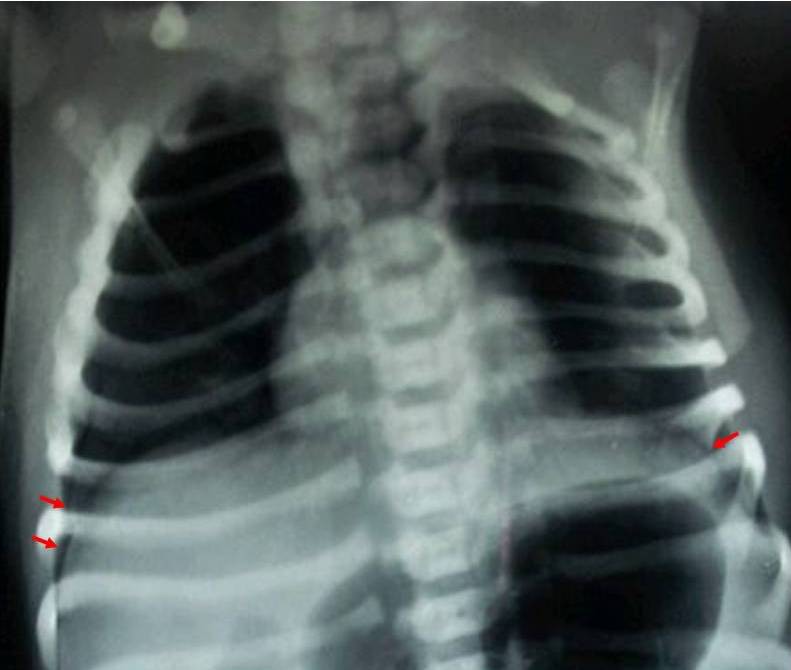
Figure 1:Chest X-ray (AP view) showing bilateral pneumothoraces

The oxygen flow increased to 100% (via oxygen hood). Oro-gastric tube feeding with mother’s milk was continued along with continuous monitoring of the clinical condition. Respiratory distress decreased within 24 hours. Gradually the FiO2 was decreased, and she was put off the oxygen after 72 hours. She did not require any surgical intervention in form of needle thoracocentesis or chest tube placement. Repeat chest radiograph done after 24 hours showed resolution of pneumothoraces (Fig. 2).

**Figure:**
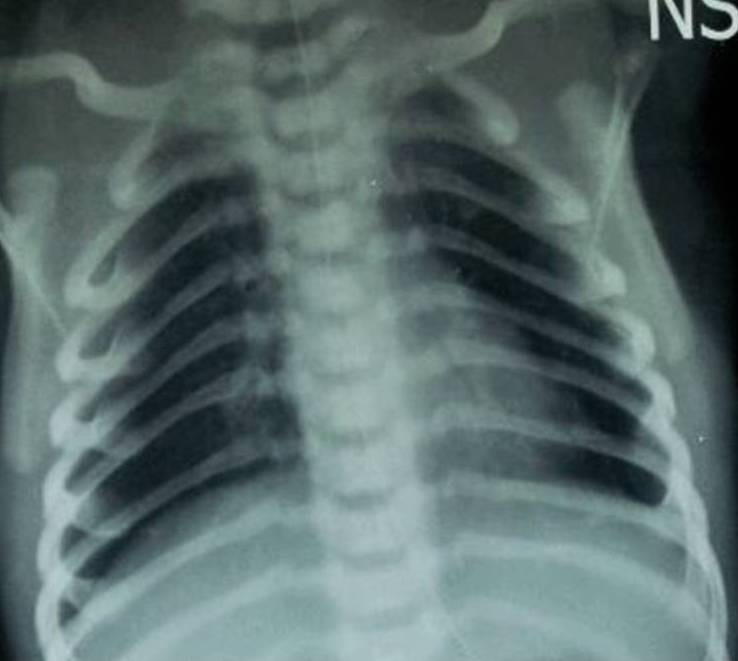
Figure 2:Chest X-ray (AP view) showing resolution of bilateral pneumothoraces

## DISCUSSION

Pneumothorax can cause neonatal respiratory distress. It can occur spontaneously or secondary to respiratory distress syndrome, aspiration of meconium, etc. The incidence of pneumothorax is 1 to 2% in term newborns.[1,2] It increased to about 6% in premature babies because of poor lung compliance.[3] In one study the frequency of pneumothorax was found to be 3 per 1000 live births.[4]

Spontaneous pneumothorax at birth results either from rupture of alveoli secondary to high pressure needed to expand previously uninflated lungs or from uneven distribution of inflating pressures among alveoli. In familial cases of spontaneous pneumothorax, folliculin gene disorders or α1-antitrypsin deficiency should be ruled out.[5] Sometimes cystic fibrosis may present with bilateral spontaneous pneumothorax during newborn period.[6]

Administration of high flow or 100% oxygen (nitrogen washout therapy) accelerates the resolution of pneumothorax.[7] To conclude, all cases of pneumothorax in newborns do not require intercostal drain. Newborn with spontaneous pneumothorax having mild or moderate distress may recover completely with no treatment other than observation in an oxygen-enriched atmosphere. For prolonged treatment (> 48 hours) with oxygen, a lower FiO2 (40%-60%) can be used safely without causing much harm.

## Footnotes

**Source of Support:** Nil

**Conflict of Interest:** None declared

